# Male and Female Subpopulations of *Salix viminalis* Present High Genetic Diversity and High Long-Term Migration Rates between Them

**DOI:** 10.3389/fpls.2016.00330

**Published:** 2016-03-18

**Authors:** Feifei Zhai, Jinmei Mao, Junxiang Liu, Xiangyong Peng, Lei Han, Zhenyuan Sun

**Affiliations:** ^1^State Key Laboratory of Tree Genetic and Breeding; Research Institute of Forestry, Chinese Academy of ForestryBeijing, China; ^2^Key Laboratory of Tree Breeding and Cultivation, State Forestry AdministrationBeijing, China; ^3^Research Institute of Economic Forest, Xinjiang Academy of ForestryXinjiang, China

**Keywords:** dioecy, *Salix viminalis*, genetic diversity, genetic differentiation, migration rate

## Abstract

Dioecy distributed in 157 flowering plant families and 959 flowering plant genera. Morphological and physiological differences between male and female plants have been studied extensively, but studies of sex-specific genetic diversity are relatively scarce in dioecious plants. In this study, 20 SSR loci were employed to examine the genetic variance of male subpopulations and female subpopulations in *Salix viminalis*. The results showed that all of the markers were polymorphic (N_a_ = 14.15, H_e_ = 0.7566) and workable to reveal the genetic diversity of *S. viminalis*. No statistically significant difference was detected between male and female subpopulations, but the average genetic diversity of male subpopulations (N_a_ = 7.12, H_e_ = 0.7071) and female subpopulations (N_a_ = 7.31, H_e_ = 0.7226) were high. Under unfavorable environments (West Liao basin), the genetic diversity between male and female subpopulations was still not significantly different, but the genetic diversity of sexual subpopulations were lower. The differentiation of the ten subpopulations in *S. viminalis* was moderate (F_ST_ = 0.0858), which was conformed by AMOVA that most of genetic variance (94%) existed within subpopulations. Pairwise F_ST_ indicated no differentiation between sexual subpopulations, which was accompanied by high long-term migrate between them (*M* = 0.73~1.26). However, little recent migration was found between sexual subpopulations. Therefore, artificial crossing or/and transplantation by cutting propagation should be carried out so as to increase the migration during the process of *ex situ* conservation.

## Introduction

Dioecious plants are not only an important part of terrestrial ecosystems, but also occupy a dominant position in forest ecosystem (Zhai and Sun, 2015). Among about 240,000 angiosperm species, 14,620 species (about 6%) are dioecy which is distributed in 157 flowering plant families and 959 flowering plant genera (Renner and Ricklefs, 1995). Morphological and physiological differences between males and females have been studied in many species, which find that the differences are more remarkable in adversity (Dawson and Ehleringer, 1993; Li et al., 2007; Zhao et al., 2011). Males and females may exhibit sexual differences because of different resource demands and allocation (Li et al., 2013). Usually, females invest more resource to reproduction, while males invest more resource in chemical or/and structural defenses (Cornelissen and Stiling, 2005). Males are often more adaptive than females in environmental stress (Dudley and Galen, 2007; Buckley and Avila-Sakar, 2013; Han et al., 2013), while some females can also be more tolerant to adversity than males (Dawson and Bliss, 1989; Montesinos et al., 2012). However, sexual differences are mainly focused on the physiological adaptation of males and females, few studies explore the genetic diversity of sexual populations at molecular genetics.

Usually, population genetic diversity can affect the adaptation and tolerance of a species, especially the genetic diversity of sexual populations (Reed and Frankham, 2003; Johansson et al., 2007; Ilves et al., 2013). Furthermore, due to that the metabolic efficiency or/and disease resistance might be increased to better afford reproduction cost, sexual genetic variation could play an important role in reproductive success (Ashman and Diefenderfer, 2001; Charpentier et al., 2005). Besides, information about the genetic diversity of males and females will increase the chances to get new genetic combination (Heikkrujam et al., 2015). Thus, assessment the genetic diversity of male and female populations is conducive to know the adaptation and evolutionary potential of a species and contributes to breed new varieties.

*Salix viminalis* is a dioecious pioneer shrub belongs to the genus *Salix*, Salicaceae. The males and females bear distinct catkins and have separate flowers which can be insects- or wind-pollinated (Karp et al., 2011). *S. viminalis* is widely distributed across the world, ranging from Atlantic Ocean eastward to Siberia and from Sweden southward to the Mediterranean Sea (Lascoux et al., 1996). In recent years, *S. viminalis* has been bred for bioenergy production owing to high biomass within short period, fast initial growth, perennial habit, repeated regrowth from coppiced stools and favorable environmental credentials (Berlin et al., 2014). Moreover, *S. viminalis* is well suited to phytoremediation of heavy mental (Klang-Westin and Perttu, 2002; Hermle et al., 2007) and organic contaminated (Ucisik and Trapp, 2006) soils. However, *S. viminalis* has not been exploited in China.

Przyborowski and Sulima (2010) have employed RAPD markers to analyze the genetic diversity and genetic similarity among 19 genotypes of *S. viminalis*, the study has identified suitable input materials for creative breeding. By using 16 isozyme loci to study the population structure of *S. viminalis* collected in Poland, Germany and Austria, it is found that the overall population differentiation is low (Lascoux et al., 1996). SSR analysis also reveals low differentiation of *S. viminalis* populations in Czech Republic, and majority of genetic variation exist within population (Trybush et al., 2012). However, as a dioecious plant, whether the genetic diversity of *S. viminalis* is related to sex has not been studied.

China, as a distribution center of willows, is rich of *S. viminalis* resources, especially in Da Hinggan Mountains. To explore the genetic diversity of males and females of *S. viminalis*, five populations (each population contains a male subpopulation and a female subpopulation) were collected in Da Hinggan Mountains, and 20 SSR markers were used to assess the genetic diversity, genetic structure and gene flow of male subpopulations and female subpopulations in *S. viminalis*.

## Materials and methods

### Sample collection and DNA extraction

**Figure 1:**
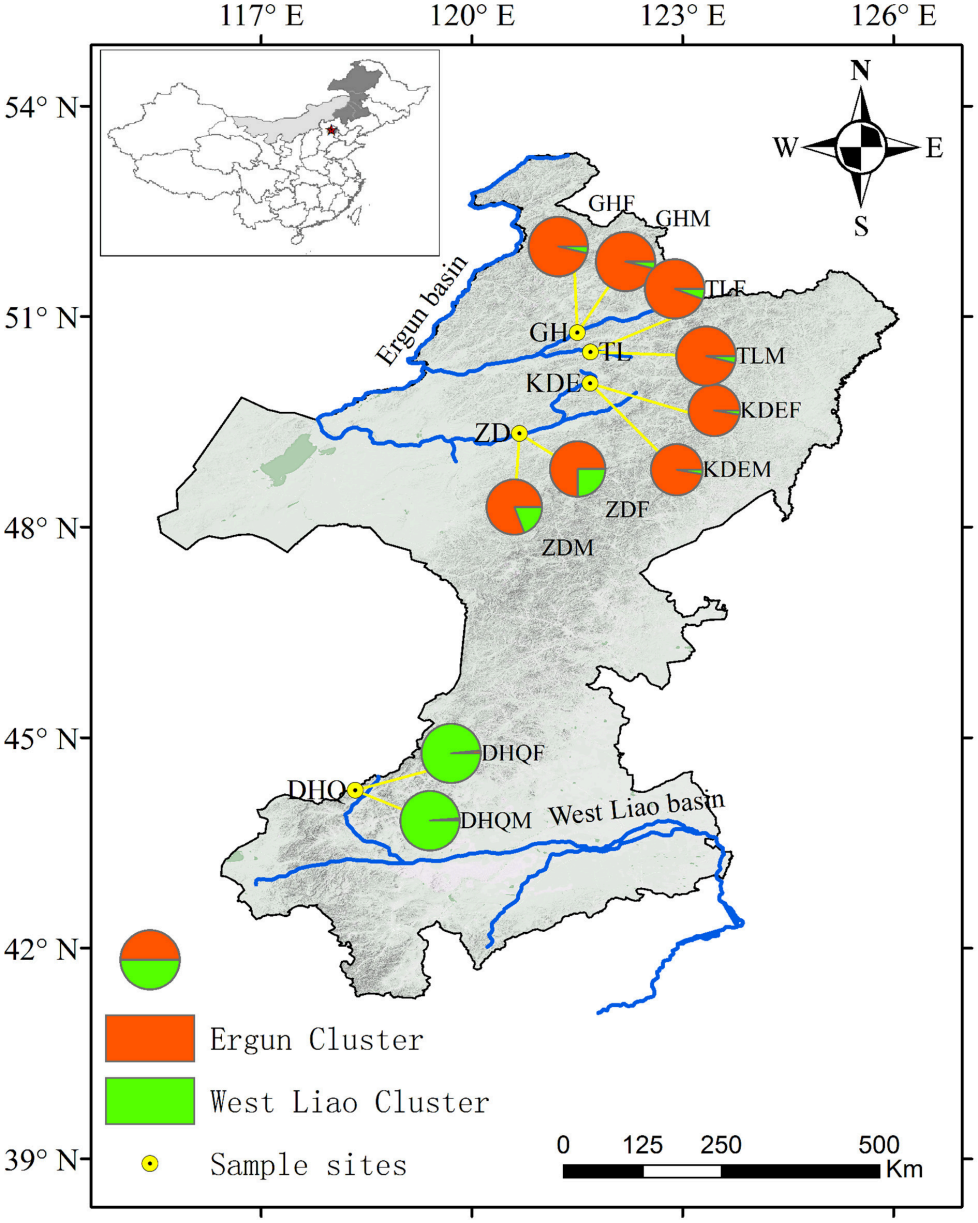
**Assignment of *S. viminalis* individuals from each subpopulation to two clusters (*K* = 2 identified by STRCUTURE)**. Pie chart sizes show the sample size of each subpopulation. Pie charts represent probability of each subpopulation belonging to each of the two clusters. Ergun cluster was shown in red color, while West Liao cluster was shown in green color.

*S. viminalis*, a diploid (2*n* = 2*x* = 38), outcrossing perennial species, which is predominantly found along streams and rivers and in other wet places. During blossom season in late April and early May in 2015, cuttings of 144 adult individuals of *S. viminalis* were collected from five populations across Ergun and West Liao basin, i.e., GH, TL, KDE, ZD, and DHQ (Figure 1, Table 1). The five populations cover an area of 3–16 km^2^ and have very high density of 60–70 no./are. Habit along Zhadun river has been effected by human activities, the distribution of *S. viminalis* are separated by road or farmland. We selected continuous distribution willow in this region as ZD population. DHQ and TL populations comprise *S. viminalis* as dominant species and *Salix linearistipularis* as associated species. The other three populations are comprised of *S. viminalis* as dominant species, *Salix schwerinii* and *S. viminalis* var. angustifolia as associated species. In every population, 13~15 male-female pairs of *S. viminalis* were randomly sampled by paired sampling method. The distance between male and female plant of each pair was controlled within 2 m, while the distances between pairs were at least 50 m apart. The males and females within a population are defined as male subpopulation and female subpopulation respectively. Each population contains a male subpopulation and a female subpopulation. After the cuttings were transported to greenhouse, they were propagated at once. One month later, fresh leaves were picked and stored at −80°C for molecular analyses. Total DNA was extracted from 150 mg fresh leaves following the modified cetyltrimethyl ammonium bromide (CTAB) protocol described by Doyle (1987). The DNA concentration was checked with a spectrophotometer (NAS-99, ACTGene Company) and the quality of DNA was verified on a 1.2% agarose gel electrophoresis.

**Table 1:** **Sampling locations of *S. viminalis* populations across Ergun and West Liao basin in China**.

**Population code**	**River**	**Basin**	**Longitude**	**Latitude**	**Altitude (m)**	**Female individual**	**Male individual**	**Population area(km^2^)**
GH	Genhe	Ergun	50°46′06.61″	121°30′09.28″	706	15	15	15
TL	Tuli	Ergun	50°29′29.73″	121°41′11.19″	734	15	15	16
KDE	Kuduer	Ergun	50°02′44.80″	121°40′55.25″	848	13	13	14
ZD	Zhadun	Ergun	49°20′03.84″	120°40′30.80″	649	14	14	3
DHQ	Dahaiqing	West Liao	44°14′48.34″	118°20′17.48″	1162	15	15	11

### SSR analysis

A total of 20 SSR makers were selected from published literatures (Barker et al., 2003; Stamati et al., 2003; Kikuchi et al., 2005; Trybush et al., 2012) which were shown in Supplementary Table 1. The PCRs was performed in a volume of 15 μl containing 6.0 μl 2.5 × PCR buffer (KAPA Taq HotStart PCR Kits, KAPA Company), 1.0 μl of each of the forward and reverse primers (5 μM), 1.0 μl of template DNA, 0.5 U of Taq DNA polymerase, and water to 15 μl. The amplifications were carried out by the following process: an initial step of 10 min at 95°C, followed by 40 cycles of 30 s at 95°C, 30 s at 58°C, 30 s at 72°C, and ending with a final extension for 7 min at 72°C. The whole cycling was conducted on a GeneAmp 9600 PCR system (Applied Biosystems). To allow multiplexing, the forward primers were fluorescently end-labeled with either 6FAM, VIC, NED or PET (Applied Biosystems). After amplification, 1 μl of PCR products were added to 0.5 μl of ROX-500 size standard (Beijing Microread Gene Technology Co., Ltd) and 8.5 μl of Hi-Di formamide (Applied Biosystems) in 96 well-plates, and denatured at 95°C for 3 min. Then the products were separated by capillary electrophoresis on 3730XL DNA analyzer (Applied Biosystems). Data were scored using the GeneMapper v 4.0 (Applied Biosystems).

### Data analysis

We applied POPGENE v 1.32 (Yeh et al., 1997) to examine genetic parameters for each locus, including number of observed alleles (N_a_), number of effective alleles (N_e_), Shannon's information index (I), observed heterozygosity (H_o_), expected heterozygosity (H_e_), inbreeding coefficient among individuals within subpopulation (F_IS_), inbreeding coefficient of an individual relative to entire population (F_IT_) and genetic differentiation among populations (F_ST_). GenAlex v 6.5 (Peakall and Smouse, 2012) was used to calculate the genetic diversity of each subpopulation, containing N_a_, N_e_, I, H_o_, H_e_ and number of private alleles (N_p_). Besides, statistical tests were performed on SPSS 18.0 (SPSS Inc., Chicago, IL, USA). For each genetic parameter, the differences between male and female subpopulations for both all loci and single locus were analyzed by paired-sample *t*-test with five pairs, while the differences between two basins were analyzed by one-way analysis of variance.

We used Bayesian clustering with an admixture model to analyze the genetic structure of all subpopulations by STRUCTURE v 2.3.4 (Pritchard et al., 2000; Falush et al., 2003). This clustering method applies the Markov Chain Monte Carlo (MCMC) algorithm. Fifteen independent runs were performed for each value of K ranging from 1 to 10 with a burn-in of 50,000 iterations followed by 100,000 iterations. In addition, the observed genetic variation within and among subpopulations characterized by analysis of molecular variance (AMOVA) was performed on GenAlex. Pair-wise values for genetic differentiation (F_ST_) were calculated by the AMOVA routine in GenAlex. Permutation procedures (9999 replicates) were used to test the significance of the differentiation between pairs of subpopulations.

Long-term effective population sizes and migration rates were estimated with MIGRATE-N v 3.4.2 (http://popgen.sc.fsu.edu/Migrate/Migrate-n.html). MIGRATE- N estimates the long- term effective population size (N_e_) of each subpopulation as parameter θ (that is 4N_e_μ, where μ is mutation rate per site). Long-term migration rates, M, 4N_e_ generations in the past based on a coalescent approach, were estimated using the maximum-likelihood mode. We adopted Brownian motion model and the Markov Chain was conducted with 10 short chains of 500 and three long chains of 2000, with an increment of 20. The burn-in at the beginning was 1000. Recent migration rates that occurred in the last few generations were performed on BAYESASS v 1.3 (Wilson and Rannala, 2003) which relies on Markov chain Monte Carlo techniques. The run consisted of 3 × 10^6^ iterations with a sampling frequency of 2000, and the first 1 × 10^6^ steps were discarded.

## Results

### The polymorphism of 20 SSR loci in *S. viminalis*

The 20 microsatellite loci used in this study were moderate to high polymorphism and a total of 283 alleles were detected across 144 *S. viminalis* individuals with an average of 14.15 observed alleles (N_a_) per locus. The average effective allele (N_e_) per locus was 5.7645. The Shannon's information index (I) ranged from 0.7084 to 3.1636 with an average of 1.8621. The mean observed heterozygosity (H_o_) and expected heterozygosity (H_e_) were 0.6542 and 0.7566 respectively. Higher H_e_ values than H_o_ at 17 loci except SB430, SB984 and SB1148 indicated heterozygote deficiencies of these loci, which were conformed by F_IT_ values (Supplementary Table 1).

### Genetic diversity of male and female subpopulations

The genetic diversity of 10 subpopulations of *S. viminalis* is shown in Table 2. Although the average genetic diversity and private alleles of female subpopulations (N_a_ = 7.3100, H_e_ = 0.7226, N_*p*_ = 3.40) was slightly higher than that of male subpopulations (N_a_ = 7.1200, H_e_ = 0.7071, N_p_ = 2.20; Table 2), the difference was not statistically significant by paired-sample *t*-test (Supplementary Table 2). Furthermore, statistical differences of male and female subpopulations at each locus were also analyzed. It was showed that N_a_, N_e_, and I of female subpopulations were significantly higher than that of male subpopulations at loci of SB1185 and SB24^*^, while H_e_ of female subpopulations was only significantly higher than male subpopulations at locus of SB24^*^ (Supplementary Table 3). Moreover, that H_o_ was lower than H_e_ in nine subpopulations except DHQF indicated heterozygote deficiency for the nine subpopulations, which was accompanied by positive fixation index (F_IS_; Table 2). In addition, the genetic parameters of subpopulations from Ergun basin were significantly higher than that from West Liao basin (Supplementary Table 4).

**Table 2:** **Genetic diversity of male and female subpopulations in *S. viminalis***.

**Sex**	**Subpopulation code**	**N_a_**	**N_e_**	**I**	**H_*o*_**	**H_e_**	**F_IS_**	**N_p_**
Male	DHQM	5.3000	2.8265	1.1308	0.5500	0.5692	−0.0009	0
	GHM	7.7000	4.8264	1.5957	0.6500	0.7313	0.0672	2
	KDEM	7.0500	4.4878	1.5578	0.7115	0.7448	−0.0015	2
	TLM	8.1500	4.9704	1.6586	0.6333	0.7572	0.1296	7
	ZDM	7.4000	4.1888	1.5666	0.7000	0.7331	0.0026	0
	Mean	7.1200	4.2600	1.5019	0.6490	0.7071	0.0394	2.20
Female	DHQF	5.3000	2.7157	1.1555	0.5900	0.5862	−0.0560	1
	GHF	7.8000	5.0060	1.6426	0.6567	0.7547	0.0829	3
	KDEF	7.5500	5.0398	1.6737	0.7308	0.7792	0.0279	3
	TLF	8.7000	5.5911	1.7486	0.6800	0.7722	0.0786	5
	ZDF	7.2000	3.9698	1.5220	0.6607	0.7206	0.0561	5
	Mean	7.3100	4.4645	1.5485	0.6636	0.7226	0.0379	3.40

N_a_, Number of observed alleles; N_e_,Number of effective alleles; I, Shannon's information index; H_o_,Observed heterozygosity; H_e_,Expected heterozygosity; F_IS_,Inbreeding coefficient among individuals within subpopulation; N_p_, Number of private alleles.

### Genetic structure and differentiation of male and female subpopulations

A clustering carried with STRUCTURE software supported an optimal value of K to be *K* = 2 for both log-likelihood values described by Pritchard et al. (2000) and ▵K values following Evanno et al. (2005) (Figure 1, Supplementary Figure 1). One of the clusters (the “West Liao cluster”) is represented by individuals from two subpopulations in West Liao basin (DHQF and DHQM). The other cluster (the “Ergun cluster”) is represented by individuals from Ergun basin.

The overall genetic differentiation was moderate with a mean F_ST_ value of 0.0858 (Supplementary Table 1). Analysis of Molecular Variance (AMOVA; Table 3) also revealed that the largest proportion of total variance (94%) existed within subpopulations and 6% was attributable to differences among subpopulations. In addition, hierarchical AMOVA was conducted based on two basins (clustering results). For two basins, 11% of the total genetic variance was ascribed to differences between basins, 1% was attributed to differences among subpopulations within basins, and the remaining 87% existed within subpopulations (Table 3). Pairwise F_ST_ values (Supplementary Table 5) revealed no statistically significant differentiation between male and female subpopulation of the same population. The differentiation was statistically significant between subpopulations from West Liao basin and that from Ergun basin at significant level of 0.001 (F_ST_ range from 0.099 to 0.162). Within Ergun basin, both ZDM and ZDF showed statistically significant differentiation with other subpopulations at level of 0.001 or 0.05 (F_ST_ range from 0.023 to 0.043). However, no statistically significant differentiation was found among subpopulations of GH, KDE, and TL.

**Table 3:** **Analysis of molecular variance (AMOVA) of ten subpopulations in *S. viminalis* by 20 SSR loci**.

**Source of variation**	**Degrees of freedom**	**Sum of squares**	**Mean squares**	**Estimated variance**	**Explained variance (%)**
**TEN SUBPOPULATIONS**					
Among subpopulations	9	188.498	20.944	0.458	6
Within subpopulations	278	1983.033	14.311	7.156	94
**TWO BASINS**					
Between basins	1	99.243	99.243	0.926	11
Among subpopulations within basins	8	89.255	11.157	0.118	1
Within subpopulations	278	1983.033	14.311	7.156	87

### Gene flow between male and female subpopulations

**Figure 2:**
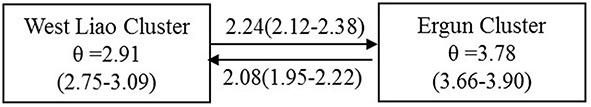
**Graphical representation for results of the migrations estimated using MIGATE-N among two basins**. Maximum-likelihood estimates and 95% confidence intervals (in parentheses) of the long-term migration rate (M) and mutation-scaled effective population size (θ) are shown.

Long-term effective population size (θ) values for subpopulations from Ergun basin were bigger ranging from 1.89 (KDEM) to 2.71(TLM), while that for DHQF and DHQM from West Liao basin were smaller which were 0.98 and 0.85. Long-term migration rates (M) ranged from 0.38 (M_DHQF → GHF_) to 1.74 (M_TLF → GHM_). That the M values between male and female subpopulations of the same population were > 0.8 (except M_GHF → GHM_) indicated high levels of historical gene exchange. There was no significant asymmetrical migration among male and female subpopulations according to overlapping 95% CIs for estimates of M in both directions [for example, M_GHF → DHQM_ = 0.80, 95% CIs = (0.68, 0.94); M_DHQM → GHF_ = 0.90, 95% CIs = (0.78, 1.03); Supplementary Table 6]. Moreover, we also estimated bidirectional gene flow between two clusters (basins) and the results were shown in Figure 2. The gene flow between the two basins was higher (2.24 and 2.08 respectively). However, recent migration among subpopulations estimated by BAYESASS was low and most of it overlapped 0 at the 95% CIs indicating little or no contemporary migration (Supplementary Table 7).

## Discussion

### High genetic diversity of male and female subpopulations

Although the average genetic diversity of female subpopulations (N_*a*_ = 7.3100, H_e_ = 0.7226) and that of male subpopulations (N_a_ = 7.1200, H_e_ = 0.7071) were both high, the genetic diversity of sexual subpopulations was not statistically significant. This was consistent with the study of *Myrica rubra* that no significant difference was found between males and females (Jia et al., 2015). Male subpopulations possess higher genetic diversity than female subpopulations in *Hippophae rhamnoides* (Chen et al., 2008) and *Pistacia atlantica* (Nosrati et al., 2012), but no statistical tests were conducted in these studies. Dioecious plants have evolved mainly to avoid inbreeding (Ainsworth, 2000), the genetic diversity and heterozygosity of dioecious plants, such as *Populus euphratica* (Wang et al., 2011), *Populus tremuloides* (Callahan et al., 2013), *Myrica rubra* (Jia et al., 2015), *Juniperus thurifera* (Teixeira et al., 2014), and *Corylus mandshurica* (Zong et al., 2015), are always higher due to obligate outbreeding. However, there are some notable exceptions. For example, *Pyrus calleryana* presents a high genetic diversity with N_a_ = 9.536 and H_e_ = 0.639 (Liu et al., 2012).

Genetic diversity is required for populations to adapt environment change, and high genetic diversity contributes to stable evolution and extension of distribution range for a species (Shen and Liu, 2001; Frankham et al., 2002). Although skewed sex ratios presented in populations of *S. viminalis* (with an overall male to female ratio of 1:5), high genetic diversity of male and female subpopulations indicated high stability of populations and this species can also maintain high evolutionary potential over long term. High genetic diversity of subpopulations further explained the extensive distribution of *S. viminalis*. In addition, lower H_o_ than H_e_ and positive F_IS_ values in nine subpopulations indicated a deficiency of heterozygotes for these subpopulations. Such a deficit might be explained by mating among close kin individuals of the opposite sex in *S. viminalis* (Young et al., 1996; Lowe et al., 2005). In previous studies of *Salicaceae* family, high positive values of inbreeding coefficients are also observed (Stamati et al., 2007; Lee et al., 2011; Perdereau et al., 2014).

Furthermore, we found that the genetic diversity of subpopulations from Ergun basin was significantly higher than that from West Liao basin. Unfavorable environments may lead to an increase in vegetative reproduction and a decrease in resource-demanding sexuality and thus the genetic variance is lost (Chen et al., 2008). Desiccation in spring and high temperature in summer for Dahaiqing river (West Liao basin) might lead to a lower genetic variability in this study. The males and females of some species behave differently to harsh environments in physiology (Xu et al., 2008; Montesinos et al., 2012), but the unfavorable environment did not lead to differences in genetic diversity between males and females of *S. viminalis*. This might be due to SSR markers are more stable than physiological trains, and current ecological environment caused no selection pressures for male and female subpopulations. The geographic explanation might be that West Liao basin could be regarded as peripheral distribution of *S. viminalis*. Toward the distributional edge, marginal populations are often genetically depauperate caused by chronic genetic drift and low gene flow (Dostálek et al., 2014) and the opportunity to communication with other populations would reduce (Wang et al., 2014), so they tend to present lower genetic diversity (Keller et al., 2011; Jiang et al., 2015). The lowest genetic diversity of male and female subpopulation appeared in Zhadun river (ZDF and ZDM), which would be influenced by insect attack, such as *Aphrophora intermedia*. It has been found that *Pinus monticola* populations under higher disease pressure possess lower genetic diversity (Kim et al., 2003, 2011). Another reason for the lower genetic diversity might be the interference of human activities.

### No genetic differentiation, high long-term migration rates, and low recent migration rates between male-female subpopulations

Microsatellite analysis revealed moderate differentiation (F_ST_ = 0.0858) of the 10 subpopulations in *S. viminalis* (Supplementary Table 1). The degree of differentiation was higher than *S. viminalis* populations in Czech Republic (F_ST_ = 0.050; Trybush et al., 2012), but was in accordance with our study (F_ST_ = 0.0761) in China (unpublished). Moderate differentiation was also conformed by AMOVA that higher level of genetic variance (94%) was found within subpopulations rather than among subpopulations (6%). This might be related to the life cycle and breeding system of *S. viminalis*. In general, the differentiation among populations of long-lived woody, wind-pollinated, out-crossed and dioecious species was significantly low (Loveless and Hamrick, 1984; Hamrick et al., 1992; Amos and Harwood, 1998). In addition, no statistically significant differentiation was found between male-female subpopulations, suggesting male-female subpopulations may share common ancestors and co-evolved. Dioecious plants have probably evolved from hermaphrodite (Barrett, 2002), the lower differentiation of male and female subpopulations might indicate that sex differentiation occurred earlier than genetic differentiation (Jia et al., 2015).

Bayesian analysis identified two putative clusters that were geographically structured and the subpopulation structure had little relationship with sex, which was consistent with previous research in *H. rhamnoides* (Chen et al., 2008). But for *Myrica rubra*, the male and female accessions can be divided into two sexual clusters although the genetic structure was low (Jia et al., 2015). Relatively high long-term migration rates (M) between two basins might reflect a historically more continuous species distribution (Bossart and Pashley, 1998). Moreover, high M values between pairs of male-female subpopulation (with *M*-values were >0.8) further explained no significant differentiation between them. As a fundamental microevolutionary force, gene flow underlines genetic differentiation among populations and influences the maintenance of genetic diversity within a species (Slatkin, 1994). Moreover, recent gene flow is of fundamental importance for analyzing the future genetic structure (Yao et al., 2007). In this study, little recent migration rates were detected between sexual subpopulations. This might be due to the inbreeding among kin restricting current seed/pollen dispersal of *S. viminalis* (Frankham et al., 2002), and the density effects of pollination services with a high density of adults can also promote short-distance pollen dispersal (inbreeding; Lowe et al., 2005; Yao et al., 2011).

In conclusion, although the genetic diversity of male and female subpopulations was high, there were no significant differences between them. Under less favorable environment in West Liao basin, the genetic diversity of male and female subpopulations was lower but still not significantly different. The subpopulations were geographically structured and had little relationship with sex. Moderate differentiation was detected among subpopulations of *S. viminalis*, but the differentiation between male-female subpopulations was not statistically significant, which was supported by high long-term migrate. However, recent migration between sexual subpopulations was low. Considering that the majority of genetic variance was within subpopulations in *S. viminalis*, so populations with higher genetic diversity and more number of private alleles should be gave priority to protection. During the process of *ex situ* conservation, artificial crossing or/and transplantation by cutting propagation should be carried out in order to increase the migration.

## Author contributions

FZ and ZS designed the study. FZ, JM, JL, XP carried out the experiments. FZ, JM, LH, and ZS analyzed the data. FZ and JM wrote the manuscript and all authors approved the final version to be published.

### Conflict of interest statement

The authors declare that the research was conducted in the absence of any commercial or financial relationships that could be construed as a potential conflict of interest.

## Supplementary material

Supplementary Figure 1 **Estimation of subpopulation structure based on Bayesian structuring by STRUCTURE vchsdateIsROCDateFalseIsLunarDateFalseDay30Month12Year18992.3.4. (A)** Mean log-likelihood value (±SD) over each *K*-value. **(B)** Delta *K*-value for each *K*-value. **(C)** Histogram of structure analysis for the model with *K* = 2.

Supplementary Table 1 **Genetic diversity and F-statistics of *S. viminalis* for 20 SSR loci**.

Supplementary Table 2 ***P*-values of five genetic parameters between male and female subpopulations by paired-sample *t*-test**.

Supplementary Table 3 ***P*-values of five genetic parameters between male and female subpopulations for 20 loci by paired-sample *t*-test**.

Supplementary Table 4 ***P*-values of five genetic parameters between two basins by one-way analysis of variance**.

Supplementary Table 5 **Pairwise F_ST_ values among ten male and female subpopulations**.

Supplementary Table 6 **Maximum-likelihood estimates and 95% confidence intervals (in parentheses) of the long-term migration rate (M) and mutation-scaled effective population size (**θ**, square brackets) using the MIGRATE-N program are shown**. Source localities are given in rows, recipient localities are in columns.

Supplementary Table 7 **The mean migration rate and (95% confidence interval) for a pair of subpopulations using by BAYESASS software are shown**. Source localities are given in columns, recipient localities are in rows. The mean and 95% confidence interval for the non-migration rates are 0.833 (0.675, 0.992) and the migration rate mean and 95% confidence interval for data sets with 10 populations are 0.0185 (3.12E-11, 0.121).
