# Supplementary figures and images for: Male and Female Subpopulations of Salix viminalis Present High Genetic Diversity and High Long-Term Migration Rates between Them
Source: Front Plant Sci. 2016 Mar 18;7:330. doi: 10.3389/fpls.2016.00330 (PMC4796010; doi:10.3389/fpls.2016.00330)

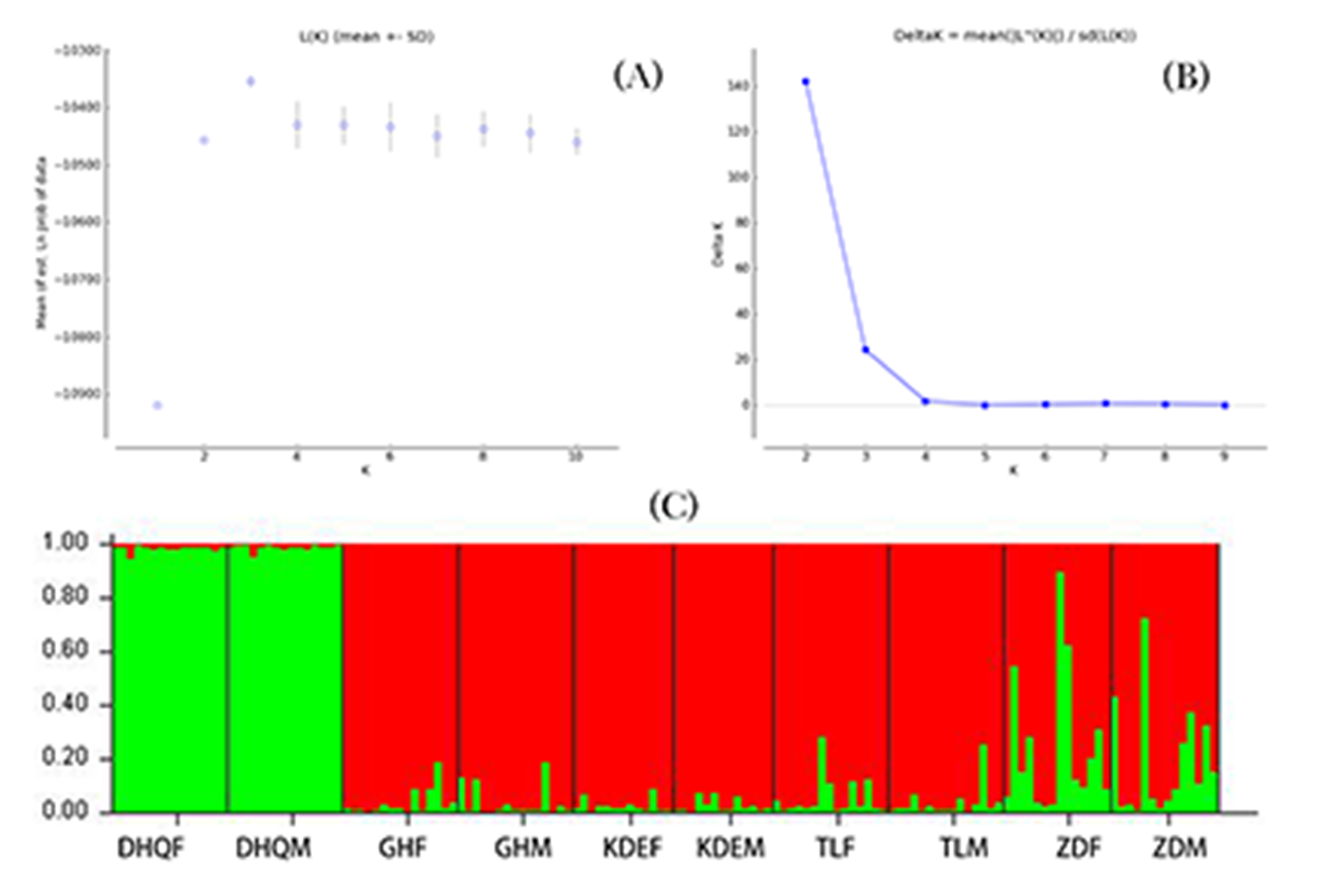

Supplement: Supplementary Figure 1 — Estimation of subpopulation structure based on Bayesian structuring by STRUCTURE vchsdateIsROCDateFalseIsLunarDateFalseDay30Month12Year18992.3.4. (A) Mean log-likelihood value (±SD) over each K-value. (B) Delta K-value for each K-value. (C) Histogram of structure analysis for the model with K = 2. [file Image1.TIF]
