# Supplementary material for: Male and Female Subpopulations of Salix viminalis Present High Genetic Diversity and High Long-Term Migration Rates between Them
Source: Front Plant Sci. 2016 Mar 18;7:330. doi: 10.3389/fpls.2016.00330 (PMC4796010; doi:10.3389/fpls.2016.00330)
Supplement: Supplementary Table 1 — Genetic diversity and F-statistics of S. viminalis for 20 SSR loci. [file Table1.DOC]

Supplementary Table 1 Genetic diversity and F-statistics of *S. viminalis* for 20 SSR loci

| Locus | N_a_ | N_e_ | I | H_o_ | H_e_ | F_IS_ | F_IT_ | F_ST_ |
| --- | --- | --- | --- | --- | --- | --- | --- | --- |
| SB38 | 14 | 4.6369 | 1.7765 | 0.7083 | 0.7871 | -0.0299 | 0.0960 | 0.1222 |
| SB430 | 9 | 4.0579 | 1.6173 | 0.7708 | 0.7562 | -0.0676 | -0.0236 | 0.0412 |
| SB288 | 30 | 11.9344 | 2.8116 | 0.8819 | 0.9194 | -0.0318 | 0.0357 | 0.0655 |
| SB1172 | 17 | 3.9288 | 1.8823 | 0.7014 | 0.7481 | -0.0069 | 0.0545 | 0.0610 |
| SB617 | 5 | 2.7324 | 1.1448 | 0.5972 | 0.6362 | -0.0821 | 0.0562 | 0.1278 |
| SB896 | 16 | 6.7732 | 2.1741 | 0.8472 | 0.8553 | -0.0729 | 0.0024 | 0.0701 |
| SB1185 | 15 | 6.5486 | 2.1392 | 0.4861 | 0.8502 | 0.3350 | 0.4268 | 0.1381 |
| SB1324 | 11 | 5.0551 | 1.9658 | 0.6944 | 0.8050 | 0.0375 | 0.1359 | 0.1022 |
| SB392 | 14 | 7.7200 | 2.2629 | 0.7917 | 0.8735 | -0.0129 | 0.0857 | 0.0974 |
| SB984 | 10 | 1.9341 | 1.1147 | 0.4931 | 0.4847 | -0.0900 | -0.0241 | 0.0604 |
| SB355 | 17 | 5.2777 | 2.1085 | 0.6389 | 0.8133 | 0.1670 | 0.2131 | 0.0553 |
| SB1366 | 9 | 4.5851 | 1.7141 | 0.4861 | 0.7846 | 0.2619 | 0.3739 | 0.1518 |
| SB1148 | 3 | 2.1600 | 0.8544 | 0.5556 | 0.5389 | -0.1046 | -0.0347 | 0.0632 |
| SB565 | 11 | 1.9627 | 1.1255 | 0.4583 | 0.4922 | 0.0021 | 0.0619 | 0.0599 |
| SB800 | 16 | 9.0808 | 2.4490 | 0.8403 | 0.8930 | -0.0006 | 0.0540 | 0.0546 |
| gSIMCT052 | 37 | 17.3523 | 3.1636 | 0.8264 | 0.9457 | 0.0625 | 0.1212 | 0.0626 |
| SB24* | 10 | 5.7512 | 1.8834 | 0.7917 | 0.8290 | -0.0127 | 0.0371 | 0.0491 |
| SB100* | 12 | 5.5712 | 1.9534 | 0.4236 | 0.8234 | 0.4134 | 0.4831 | 0.1188 |
| Shuk058 | 4 | 1.8117 | 0.7084 | 0.3889 | 0.4496 | 0.0251 | 0.1346 | 0.1123 |
| Shuk124 | 23 | 6.4238 | 2.3930 | 0.7014 | 0.8473 | 0.0716 | 0.1653 | 0.1009 |
| Mean | 14.15 | 5.7649 | 1.8621 | 0.6542 | 0.7566 | 0.0489 | 0.1305 | 0.0858 |

Note: N_a_=Number of observed alleles; N_e_=Number of effective alleles; I= Shannon’s information index; H_o_=Observed heterozygosity; H_e_=Expected heterozygosity; F_IS_=Inbreeding coefficient among individuals within subpopulation; F_IT_=Inbreeding coefficient of an individual relative to entire population; F_ST_=Genetic differentiation among populations.
