# Supplementary material for: Male and Female Subpopulations of Salix viminalis Present High Genetic Diversity and High Long-Term Migration Rates between Them
Source: Front Plant Sci. 2016 Mar 18;7:330. doi: 10.3389/fpls.2016.00330 (PMC4796010; doi:10.3389/fpls.2016.00330)
Supplement: Supplementary Table 2 — P-values of five genetic parameters between male and female subpopulations by paired-sample t-test. [file Table2.DOC]

Supplementary Table 2 P values of five genetic parameters between male and female subpopulations by paired-sample t test

|  | N_a_ | N_e_ | I | H_o_ | H_e_ |
| --- | --- | --- | --- | --- | --- |
| P value | 0.2610 | 0.2937 | 0.1694 | 0.3909 | 0.1173 |
