# Supplementary material for: Male and Female Subpopulations of Salix viminalis Present High Genetic Diversity and High Long-Term Migration Rates between Them
Source: Front Plant Sci. 2016 Mar 18;7:330. doi: 10.3389/fpls.2016.00330 (PMC4796010; doi:10.3389/fpls.2016.00330)
Supplement: Supplementary Table 3 — P-values of five genetic parameters between male and female subpopulations for 20 loci by paired-sample t-test. [file Table3.DOC]

Supplementary Table 3 P values of five genetic parameters between male and female subpopulations for 20 loci by paired-sample t test

| Locus | N_a_ | N_e_ | I | H_o_ | H_e_ |
| --- | --- | --- | --- | --- | --- |
| SB38 | 1.0000 | 0.3642 | 0.5348 | 0.3504 | 0.2105 |
| SB430 | 0.3739 | 0.2978 | 0.3384 | 0.2815 | 0.3416 |
| SB288 | 0.3375 | 0.2989 | 0.3360 | 0.7040 | 0.2995 |
| SB1172 | 0.5012 | 0.4330 | 0.5016 | 0.8907 | 0.5851 |
| SB617 | 1.0000 | 0.8957 | 0.6543 | 0.8724 | 0.6359 |
| SB896 | 1.0000 | 0.8510 | 0.7655 | 0.6646 | 0.5654 |
| SB1185 | 0.0086 | 0.0494 | 0.0249 | 0.1141 | 0.1151 |
| SB1324 | 0.2420 | 0.9778 | 0.5049 | 0.9531 | 0.4187 |
| SB392 | 0.3046 | 0.4304 | 0.4648 | 0.0965 | 0.9631 |
| SB984 | 0.4766 | 0.6773 | 0.8057 | 0.2325 | 0.4125 |
| SB355 | 1.0000 | 0.1048 | 0.0457 | 0.7406 | 0.1180 |
| SB1366 | 0.8276 | 0.4983 | 0.5526 | 0.8711 | 0.4914 |
| SB1148 | 0.6213 | 0.4349 | 0.6854 | 0.3906 | 0.3923 |
| SB565 | 0.8541 | 0.4184 | 0.8666 | 0.1827 | 0.7961 |
| SB800 | 0.4954 | 0.2013 | 0.1840 | 0.5695 | 0.1315 |
| gSIMCT052 | 0.4766 | 0.0279 | 0.0815 | 0.8733 | 0.1228 |
| SB24* | 0.0161 | 0.0040 | 0.0007 | 0.9559 | 0.0010 |
| SB100* | 0.2080 | 0.3651 | 0.3660 | 0.2199 | 0.8397 |
| Shuk058 | 0.1778 | 0.8564 | 0.9065 | 0.9444 | 0.7712 |
| Shuk124 | 0.3739 | 0.3873 | 0.1894 | 0.4640 | 0.1728 |
