# Supplementary material for: Male and Female Subpopulations of Salix viminalis Present High Genetic Diversity and High Long-Term Migration Rates between Them
Source: Front Plant Sci. 2016 Mar 18;7:330. doi: 10.3389/fpls.2016.00330 (PMC4796010; doi:10.3389/fpls.2016.00330)
Supplement: Supplementary Table 4 — P-values of five genetic parameters between two basins by one-way analysis of variance. [file Table4.DOC]

Supplementary Table 4 P values of five genetic parameters between two basins by one-way analysis of variance

|  | N_a_ | N_e_ | I | H_o_ | H_e_ |
| --- | --- | --- | --- | --- | --- |
| P value | 0.0003 | 0.0009 | 0.0000 | 0.0033 | 0.0000 |
