# Supplementary material for: Male and Female Subpopulations of Salix viminalis Present High Genetic Diversity and High Long-Term Migration Rates between Them
Source: Front Plant Sci. 2016 Mar 18;7:330. doi: 10.3389/fpls.2016.00330 (PMC4796010; doi:10.3389/fpls.2016.00330)
Supplement: Supplementary Table 5 — Pairwise FST values among ten male and female subpopulations. [file Table5.DOC]

Supplementary Table 5 Pairwise F_ST_ values among ten male and female subpopulations

|  | DHQF | DHQM | GHF | GHM | KDEF | KDEM | TLF | TLM | ZDF | ZDM |
| --- | --- | --- | --- | --- | --- | --- | --- | --- | --- | --- |
| DHQF | -- |  |  |  |  |  |  |  |  |  |
| DHQM | 0.010^NS^ | -- |  |  |  |  |  |  |  |  |
| GHF | 0.137** | 0.149** | -- | -- |  |  |  |  |  |  |
| GHM | 0.149** | 0.156** | 0.000^NS^ | -- |  |  |  |  |  |  |
| KDEF | 0.134** | 0.143** | 0.007^NS^ | 0.006^NS^ | -- |  |  |  |  |  |
| KDEM | 0.151** | 0.162** | 0.009^NS^ | 0.006^NS^ | 0.002^NS^ | -- |  |  |  |  |
| TLF | 0.127** | 0.127** | 0.000^NS^ | 0.000^NS^ | 0.002^NS^ | 0.014* | -- |  |  |  |
| TLM | 0.134** | 0.152** | 0.005^NS^ | 0.000^NS^ | 0.003^NS^ | 0.009^NS^ | 0.002^NS^ | -- |  |  |
| ZDF | 0.099** | 0.111** | 0.029** | 0.039** | 0.023* | 0.034* | 0.034** | 0.032** | -- |  |
| ZDM | 0.117** | 0.129** | 0.034** | 0.041** | 0.023* | 0.043** | 0.033** | 0.035** | 0.000^NS^ | -- |

Note: **P*<0.05; ***P*<0.001 Probability values are based on 9999 permutations.
