# Supplementary material for: Male and Female Subpopulations of Salix viminalis Present High Genetic Diversity and High Long-Term Migration Rates between Them
Source: Front Plant Sci. 2016 Mar 18;7:330. doi: 10.3389/fpls.2016.00330 (PMC4796010; doi:10.3389/fpls.2016.00330)
Supplement: Supplementary Table 6 — Maximum-likelihood estimates and 95% confidence intervals (in parentheses) of the long-term migration rate (M) and mutation-scaled effective population size (θ, square brackets) using the MIGRATE-N program are shown. Source localities are given in rows, recipient localities are in columns. [file Table6.DOC]

Supplementary 6 Maximum-likelihood estimates and 95% confidence intervals (in parentheses) of the long-term migration rate (M) and mutation-scaled effective population size (θ, square brackets) using the MIGRATE-N program are shown. Source localities are given in rows, recipient localities are in columns.

|  | DHQF  [0.98] | DHQM  [0.85] | GHF  [2.48] | GHM  [2.26] | KDEF  [2.39] | KDEM  [1.89] | TLF  [2.54] | TLM  [2.71] | ZDF  [2.08] | ZDM  [2.34] |
| --- | --- | --- | --- | --- | --- | --- | --- | --- | --- | --- |
| DHQF | -- | 0.87  (0.75-1.01) | 0.38  (0.30-0.46) | 0.78  (0.66-0.90) | 1.17  (0.99-1.32) | 0.75  (0.65-0.87) | 1.02  (0.90-1.16) | 0.98  (0.84-1.12) | 1.04  (0.90-1.25) | 0.94  (0.81-1.08) |
| DHQM | 1.22  (1.06-1.38) | -- | 0.90  (0.78-1.03) | 1.08  (0.94-1.23) | 0.91  (0.79-1.05) | 0.74  (0.63-0.92) | 0.49  (0.41-0.59) | 1.00  (0.87-1.15) | 1.55  (1.37-1.73) | 0.83  (0.71-0.96) |
| GHF | 1.20  (0.99-1.37) | 0.80  (0.68-0.94) | -- | 0.73  (0.62-0.86) | 0.93  (0.81-1.07) | 1.38  (1.23-1.54) | 0.90  (0.78-1.03) | 1.48  (1.31-1.66) | 1.13  (0.98-1.28) | 1.11  (0.97-1.26) |
| GHM | 0.77  (0.64-0.90) | 0.99  (0.85-1.14) | 1.14  (1.01-1.28) | -- | 1.23  (1.09-1.39) | 1.13  (0.99-1.31) | 0.62  (0.50-0.72) | 1.03  (0.89-1.18) | 1.70  (1.53-1.90) | 0.94  (0.81-1.11) |
| KDEF | 0.73  (0.61-0.86) | 0.87  (0.74-1.01) | 1.01  (0.89-1.15) | 1.33  (1.14-1.49) | -- | 0.87  (0.71-1.00) | 1.37  (1.22-1.53) | 1.35  (1.14-1.52) | 1.14  (0.99-1.30) | 0.98  (0.85-1.16) |
| KDEM | 0.94  (0.80-1.12) | 1.08  (0.94-1.24) | 0.70  (0.59-0.84) | 0.94  (0.81-1.07) | 0.95  (0.82-1.12) | -- | 1.72  (1.56-1.90) | 1.24  (1.09-1.40) | 1.30  (1.11-1.47) | 1.55  (1.38-1.73) |
| TLF | 0.86  (0.73-1.03) | 0.86  (0.74-1.04) | 1.19  (1.06-1.34) | 1.74  (1.57-1.93) | 1.03  (0.87-1.17) | 1.34  (1.20-1.50) | -- | 1.13  (0.99-1.29) | 1.10  (0.96-1.26) | 0.53  (0.44-0.64) |
| TLM | 0.66  (0.52-0.79) | 0.73  (0.61-0.86) | 1.23  (1.09-1.38) | 1.21  (1.07-1.37) | 1.12  (0.98-1.31) | 0.94  (0.81-1.07) | 0.81  (0.70-0.93) | -- | 0.92  (0.79-1.07) | 1.15  (1.01-1.31) |
| ZDF | 1.11  (0.96-1.27) | 0.92  (0.79-1.06) | 0.86  (0.75-0.99) | 1.13  (0.97-1.29) | 1.10  (0.96-1.29) | 1.17  (1.04-1.32) | 1.07  (0.94-1.21) | 1.06  (0.92-1.21) | -- | 1.26  (1.11-1.47) |
| ZDM | 0.77  (0.65-0.91) | 0.83  (0.71-0.97) | 1.12  (0.99-1.29) | 0.88  (0.76-1.02) | 1.34  (1.19-1.50) | 0.71  (0.60-0.83) | 0.89  (0.77-1.01) | 1.13  (0.98-1.28) | 1.15  (1.00-1.31) | -- |
