# Supplementary material for: Male and Female Subpopulations of Salix viminalis Present High Genetic Diversity and High Long-Term Migration Rates between Them
Source: Front Plant Sci. 2016 Mar 18;7:330. doi: 10.3389/fpls.2016.00330 (PMC4796010; doi:10.3389/fpls.2016.00330)
Supplement: Supplementary Table 7 — The mean migration rate and (95% confidence interval) for a pair of subpopulations using by BAYESASS software are shown. Source localities are given in columns, recipient localities are in rows. The mean and 95% confidence interval for the non-migration rates are 0.833 (0.675, 0.992) and the migration rate mean and 95% confidence interval for data sets with 10 populations are 0.0185 (3.12E-11, 0.121). [file Table7.DOC]

Supplementary 7 The mean migration rate and (95% confidence interval) for a pair of subpopulations using by BAYESASS software are shown. Source localities are given in columns, recipient localities are in rows. The mean and 95% confidence interval for the non-migration rates are 0.833 (0.675, 0.992) and the migration rate mean and 95% confidence interval for data sets with 10 populations are 0.0185 (3.12E-11, 0.121).

|  | DHQF | DHQM | GHF | GHM | KDEF | KDEM | TLF | TLM | ZDF | ZDM |
| --- | --- | --- | --- | --- | --- | --- | --- | --- | --- | --- |
| DHQF | **0.835**  **(0.68-0.99)** | 0.019  (0-0.11) | 0.018  (0-0.12) | 0.019  (0-0.13) | 0.018  (0-0.12) | 0.019  (0-0-0.12) | 0.018  (0-0.12) | 0.020  (0-0.11) | 0.018  (0-0.12) | 0.002  (0-0.03) |
| DHQM | 0.019  (0-0.13) | **0.835**  **(0.67-0.99)** | 0.018  (0-0.11) | 0.018  (0-0.13) | 0.019  (0-0.13) | 0.016  (0-0.11) | 0.019  (0-0.12) | 0.022  (0-0.12) | 0.019  (0-0.13) | 0.002  (0-0.02) |
| GHF | 0.017  (0-0.11) | 0.018  (0-0.12) | **0.831**  **(0.68-0.99)** | 0.020  (0-0.13) | 0.018  (0-0.12) | 0.020  (0-0.13) | 0.019  (0-0.13) | 0.021  (0-0.12) | 0.019  (0-0.12 | 0.002  (0-0.02) |
| GHM | 0.020  (0-0.13) | 0.019  (0-0.12) | 0.019  (0-0.12) | **0.831**  **(0.68-99)** | 0.019  (0-0.13) | 0.018  (0-0.12) | 0.018  (0-0.12) | 0.022  (0-0.12) | 0.017  (0-0.12) | 0.002  (0-0.02) |
| KDEF | 0.018  (0-0.11) | 0.018  (0-0.12) | 0.017  (0-0.12) | 0.019  (0-0.11) | **0.835**  **(0.68-0.99)** | 0.020  (0-0.12) | 0.019  (0-0.12) | 0.022  (0-0.12) | 0.016  (0-0.11) | 0.002  (0-0.02) |
| KDEM | 0.017  (0-0.12) | 0.019  (0-0.13) | 0.019  (0-0.12) | 0.020  (0-0.13) | 0.020  (0-0.13) | **0.830**  **(0.67-0.99)** | 0.019  (0-0.12) | 0.023  (0-0.12) | 0.019  (0-0.12) | 0.002  (0-0.02) |
| TLF | 0.018  (0-0.12) | 0.017  (0-0.12) | 0.020  (0-0.12) | 0.018  (0-0.13) | 0.018  (0-0.12) | 0.019  (0-0.12) | **0.836**  **(0.67-0.99)** | 0.023  (0-0.13) | 0.019  (0-0.13) | 0.002  (0-0.02) |
| TLM | 0.020  (0-0.13) | 0.018  (0-0.12) | 0.020  (0-0.13) | 0.018  (0-0.12) | 0.017  (0-0.12) | 0.018  (0-0.11) | 0.018  (0-0.12) | **0.781**  **(0.67-0.95)** | 0.018  (0-0.11) | 0.003  (0-0.02) |
| ZDF | 0.018  (0-0.12) | 0.019  (0-0.11) | 0.019  (0-0.13) | 0.018  (0-0.12) | 0.018  (0-0.12) | 0.020  (0-0.13) | 0.017  (0-0.11) | 0.023  (0-0.12) | **0.837**  **(0.68-0.99)** | 0.022  (0-0.02) |
| ZDM | 0.019  (0-0.13) | 0.019  (0-0.12) | 0.018  (0-0.11) | 0.019  (0-0.13) | 0.017  (0-0.12) | 0.020  (0-0.12) | 0.018  (0-0.11) | 0.043  (0-0.15) | 0.018  (0-0.11) | **0.978**  **(0.93-0.99)** |
